# Effects of human umbilical cord mesenchymal stem cell-derived exosomes in the rat osteoarthritis models

**DOI:** 10.1093/stcltm/szae031

**Published:** 2024-06-24

**Authors:** Huanfeng Yang, Yiqin Zhou, Bi Ying, Xuhui Dong, Qirong Qian, Shaorong Gao

**Affiliations:** Institute for Regenerative Medicine, Shanghai East Hospital, Shanghai Key Laboratory of Signaling and Disease Research, School of Life Sciences and Technology, Tongji University, Shanghai, 200120, People’s Republic of China; Department of R&D, Oricell Therapeutics, Shanghai, 201203, People’s Republic of China; Department of Orthopedics, Shanghai Changzheng Hospital, Naval Medical University, Shanghai, 200003, People’s Republic of China; Department of R&D, Oricell Therapeutics, Shanghai, 201203, People’s Republic of China; Department of R&D, Oricell Therapeutics, Shanghai, 201203, People’s Republic of China; Department of Orthopedics, Shanghai Changzheng Hospital, Naval Medical University, Shanghai, 200003, People’s Republic of China; Institute for Regenerative Medicine, Shanghai East Hospital, Shanghai Key Laboratory of Signaling and Disease Research, School of Life Sciences and Technology, Tongji University, Shanghai, 200120, People’s Republic of China; Shanghai Key Laboratory of Maternal Fetal Medicine, Shanghai Institute of Maternal-Fetal Medicine and Gynecologic Oncology, Clinical and Translation Research Center, Shanghai First Maternity and Infant Hospital, School of Life Science and Technology, Tongji University, Shanghai, 201204, People’s Republic of China; Frontier Science Center for Stem Cell Research, Tongji University, Shanghai, 200092, People’s Republic of China

**Keywords:** mesenchymal stem cells, exosomes, osteoarthritis, umbilical cord, regeneration

## Abstract

Mesenchymal stem cells (MSCs) offer great potential for treatment of osteoarthritis (OA) by promoting articular cartilage regeneration via paracrine secretion of exosomes; however, the underlying mechanisms are not fully understood. This study aimed to explore the therapeutic effects of exosomes secreted by human umbilical cord-derived MSCs (hUC-MSCs) in rat models of OA and reveal the underlying mechanisms. UC-MSCs and UC-MSC-exosomes were prepared and identified by transmission electron microscopy and flow cytometry. IL-1β-induced OA chondrocytes and the operation and collagenase-induced OA rat models were established. The results of micro-computed tomography, histology, and immunohistochemistry showed that UC-MSC-exosomes promoted cartilage regeneration in OA rats. ELISA results showed that the levels of synovial fluid cytokines, TNF-α, IL-1β, and IL-6, were lower in exosome therapy group than control group in both OA rat models. Exosome treatment significantly downregulated the expression of MMP-13 and ADAMTS-5 in chondrocytes stimulated by IL-1β, and upregulated collagen II expression. These findings suggest that hUC-MSC-exosomes offer a promising option for the therapy for OA.

## Graphical Abstract

**Graphical Abstract:**
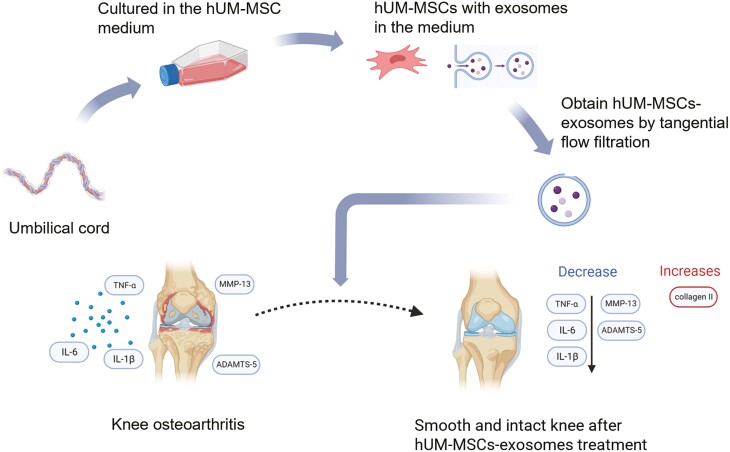
Exosomes secreted by human umbilical cord-derived mesenchymal stem cells promoted cartilage regeneration in OA rats. The levels of synovial fluid cytokines (TNF-α, IL-1β, and IL-6) were significantly reduced in the synovial fluid of exosome treated group, and exosome treatment also significantly downregulated the expression of MMP-13 and ADAMTS-5 in chondrocytes stimulated by IL-1β.

**Significance Statement**

Osteoarthritis (OA) is a prevalent degenerative joint disease that poses a substantial burden on public health and the quality of life of affected individuals. We found hUC-MSC-derived exosomes significantly promoted cartilage regeneration in OA rat models. The synovial fluid cytokines (TNF-α, IL-1β, and IL-6) and matrix-degrading enzymes (MMP-13 and ADAMTS-5 ) were deceased, while collagen II was increased after hUC-MSC-exosome therapy in OA rat models. Our findings suggest that hUC-MSC-exosomes offer a promising option for the therapy for OA.

## Introduction

Osteoarthritis (OA) is a degenerative joint disorder mainly affecting diarthrodial joints with aging and obesity as the prominent risk factors.^1^ The key pathological changes in OA are cartilage destruction, subchondral bone remodeling and resorption of bone, hypertropic differentiation of chondrocytes, neovascularization of synovial tissue, and joint cartilage calcification.^2,3^ OA is a combination of mechanical and biological factors; the physiological imbalance of extracellular matrix, chondrocytes, and subchondral bone leads to the greater catabolism of articular cartilage than anabolism.^4,5^ Systemical or local inflammation within the synovium plays important roles in the pathogenesis of OA, and proinflammatory cytokines contribute to the destruction of cartilage and synovitis, such as IL-6, IL-1β, and TNF-α.^6-9^ Increased local level of matrix-degrading enzymes, such as MMP-13 and ADAMTS-5, also promote cartilage degradation in OA.^6^

Mesenchymal stem cells (MSCs) are multipotent stem cells with immunomodulatory and anti-inflammatory effects.^10^ MSCs are present in bone marrow, peripheral blood, cord blood, and adipose tissue, and have the capacity to differentiate into mesodermal lineages, such as osteoblasts, chondrocytes, and adipocytes.^11^ Based on the evidence of their pluripotent differentiation and immunomodulatory properties, MSCs have been at the center of attention in the field of regenerative medicine, especially with regard to OA.^12^ MSCs facilitate cartilage repair by promoting the proliferation and inhibiting the apoptosis of chondrocytes via paracrine secretion of exosomes.^13^

Exosomes are small lipid membrane extracellular vesicles with a diameter of 30-150 nm.^14^ Exosomes are secreted by nearly all eukaryotic cells through fusion of cytoplasmic multivesicular bodies (late endosomes) with the plasma membrane.^15^ They contain various types of molecules, including lipids, proteins, DNA, mRNA, and noncoding RNAs from parent cells, which allows them to participate in intercellular molecular exchange and communication.^16^ Exosomes secreted by human MSCs promote cartilage regeneration^17^; however, the underlying mechanisms are not fully revealed.

In this study, we investigated the therapeutic effects of exosomes secreted by human umbilical cord-derived MSCs (hUC-MSCs) in rat models of OA. We found that UC-MSC-exosomes promoted cartilage regeneration in operation and collagenase-induced OA rat models. The levels of synovial fluid cytokines, TNF-α, IL-1β, and IL-6, were lower in exosome therapy group than control group in both OA rat models. Exosome treatment significantly downregulated the expression of MMP-13 and ADAMTS-5 in chondrocytes stimulated by IL-1β, and upregulated collagen II expression. Our data suggest that hUC-MSC-exosomes may be represented as an alternative promising strategy for OA therapy.

## Materials and methods

### Human umbilical cord mesenchymal stem cell culture

Human UC-MSCs were isolated from umbilical cord, expanded in DMEM supplemented with 5% EliteGro-Adv (serum-free supplement). The fourth to sixth passages of hUC-MSCs were used in subsequent experiments. The phenotype of hUC-MSC was characterized by flow cytometry and the human MSC Analysis Kit (BD Biosciences). Adipogenesis were visualized by HCS LipidTOX Green neutral lipid stain (Thermo Fisher Scientific). Osteogenic differentiation was detected by alizarin red S staining.

### Extraction and identification of hUC-MSC-derived exosomes

Human UC-MSCs-conditioned medium was collected after 72 hours and centrifuged at 2500 × *g* for 25 minutes. The supernatants were size fractionated and concentrated 50× by tangential flow filtration using a membrane with a molecular weight cutoff of 100 kDa (Sartorius, Gottingen, Germany). The exosomes were filtered with a 0.22-µm filter (Merck Millipore, Billerica, MA, USA) and stored in −80 °C freezer until use.

The concentration and size distribution of hUC-MSC-exosomes were measured using Nanoparticle Tracking Analysis in a NanoSight LM10-12 instrument. Transmission electron microscopy (TEM) was used to observe exosome morphology. Exosomal surface marker proteins CD81, CD9, and CD63 were analyzed by flow cytometry.

### Establishment of the OA model

Sprague Dawley rats (8-week-old, 300-350 g, male) were provided by Shanghai Sinopharm Group and housed in an environment (25 °C, 70% humidity, and 12 hours of light) with free access to food and water. The experimental knee OA rat models were established by surgery or collagenase injection into the knee joint cavity.^18^ In brief, the rats were anesthetized via intraperitoneal injection of 0.3% pentobarbital sodium (10 mL/kg; Sigma). After the left knee joints of rats were opened to expose articular cavity, the anterior cruciate ligaments, medial collateral ligaments and medial menisci were cut off. For the collagenase-induced OA model, 50 μL collagenase (0.4 mg/mL, Type II, Sigma, C6885) was dissolved in 600 μL of 0.9% sterile saline, and injected into the left hind knee joint cavity of the rats. The procedure was repeated on days 4 and 7 postinjection.

The rats underwent joint cavity surgery or collagenase injection were randomly divided into 2 groups: control group with saline treatment and hUC-MSCs-EXO treatment group with 5 × 10^9^ exosomes injection.

### Micro-computed tomography

Four weeks post treatment, rat joints were scanned with the high-resolution micro-computed tomography (micro-CT) system Scanner RS-9 system (GE Healthcare) at a source voltage of 50 kV, a source current of 500 mA and using an aluminum filter of 0.5 mm. Samples were rotated by 180° with a rotation step of 0.35°, and the nominal resolution was 17.5 μm. The images were reconstructed by the software Micro View to obtain micro-CT sections with corrections for alignment beam hardening and ring artifact reduction. The cortical bone and epiphyseal trabecular bone regions were evaluated and the volumes of interest (VOI) were defined. For cortical bone, the VOI was 1 mm mediolaterally and 2 mm ventrodorsally in each femoral epicondyle or tibial plateau. For the epiphyseal trabecular bone, the VOI was defined by the anatomical borders of the femur condyle or the tibial plateau. Cortical bone thickness (Ct.Th, mm) was measured in the VOIs of epiphyseal cortical bone. Bone volume (BV/TV, %), trabecular thickness (TbTh, mm), trabecular number (Tb.N, mm^-1^) and trabecular separation (TbSp, mm) were calculated in the VOIs of epiphyseal trabecular bone. To quantify osteophytes volume, osteophytes within each contiguous coronal image section of both femurs and tibiae were identified and outlined manually.^19^

### Histology

The left knee joints were fixed in 10% buffered neutral formalin-solution for 3 days. Then, joints were rinsed in distilled water for 30 minutes and transferred to 20% EDTA solution. The solution was changed every 3 days until the decalcification is completed. Decalcified samples were dehydrated in graded alcohol solutions and embedded in paraffin. Tissue sections (5 ± 1 μm) were harvested at 30-μm intervals through the entire joint by a semiautomated microtome. Slides were stained with safranine O-/fast green, hematoxylin/eosin, and Alcian blue.

### Immunohistochemistry

Tissue sections were deparaffinizated, rehydrated in decreasing graded ethanol solutions, PBS rinsed, and immunostained. The sections were incubated with anti-Collagen I antibody (Abcam, ab34710) and anti-Collagen II antibody (Abcam, ab34712) overnight at 4 °C. The slides were washed 5 times in PBS and incubated with goat anti-rabbit IgG H&L (HRP; Abcam, ab205718) at 37 °C for 30 minutes and visualized with diaminobenzidine. Nuclei were counterstained with hematoxylin.^20^ Chondrocytes were immunostained with anti-Collagen II antibody (Abcam, ab34712).

### Enzyme-linked immunosorbent assay (ELISA)

The levels of synovial fluid cytokines including IL-6, IL-1β, and TNF-α were measured 4 weeks post the first treatment of rats with ELISA kits (Enzyme-linked Biotechnology, China) according to the manufacturer’s instructions. The absorption at 450 nm was measured with an ultraviolet microplate reader (Thermo Fisher Scientific Corporation, Massachusetts, USA).

### Isolation, culture, and treatment of chondrocytes

Chondrocytes were isolated from the knee joint of rats as described.^21^ In brief, the articular cartilage tissue of the knee joint of rat hind legs was harvested and digested with 0.2% trypsin and Type II collagenase. Cells were cultured in Dulbecco’s modified Eagle’s medium (DMEM, Gibco) plus 10% FBS. Passage1-3 chondrocytes were used for experiments. The chondrocytes (1 × 10^5^ cells per well) were placed in 24-well plates, and the medium was replaced with DMEM the following day. The chondrocytes were stimulated with recombinant human IL-1β (Sigma, H6291) for 48 hours, and treated with 200 µg/mL hUC-MSC-exosomes.

### Western blot

The rat primary chondrocytes were washed in ice-cold PBS and total proteins were extracted with the Protein Extraction kit (Nanjing KeyGen Biotech. Co., Ltd., Nanjing, China). The protein concentrations were measured with the bicinchonic acid protein assay kit (Boster Biological Technology Co., Wuhan, China). The protein samples were denatured at 95˚C for 5 minutes and separated by SDS-PAGE. The proteins were subsequently transferred to polyvinylidene difluoride membranes, which were incubated with anti-Collagen II (Abcam, ab34712), anti-MMP-13 (Abcam, ab39012) and anti-ADAMTS-5 (Abcam, ab41037) at 4 °C overnight, and goat anti-rabbit IgG H&L (HRP; Abcam, ab205718) for 2 hours. The signal was visualized with a BeyoECL Plus kit (Beyotime Institute of Biotechnology).

### Quantitative real-time reverse transcription PCR

The total RNA was extracted from rat primary chondrocytes by the RNAiso Plus, and reverse transcribed into cDNA with the PrimeScript RT reagent Kit according to the manufacturer’s instructions. Quantitative real-time reverse transcription PCR (qRT-PCR) was performed using SYBR1 Premix Ex Taq (Tli RNaseH Plus). The specific primers are shown in Supplementary Table S1. The PCR was performed using the following cycle parameters: 95 °C for 10 minutes, 40 cycles of 95 °C for 30 seconds and 60 °C for 1 minute, and a final extension of 72 °C for 5 minutes. Relative quantitative evaluation of Collagen II, MMP-13, and ADAMTS-5 was performed with the 2^−△△CT^ method and determined relative to GAPDH.

### Synovial fluid collection

All the rats were sacrificed and the knee joint was dissected. A 0.5-cm incision was cut in the suprapatellar bursa. The joint cavity was irrigated with 1 mL saline 3 times, and the irrigated saline was collected in the siliconized tube.

### Statistical analysis

The statistical analysis was carried out with GraphPad Prism (version 5.01) by paired *t* test analysis. All data were presented as means ± SD. A *P* value <.05 was considered as a statistically significant difference.

## Results

### Characterization of hUC-MSC and hUC-MSC-exosomes

**Figure 1.**
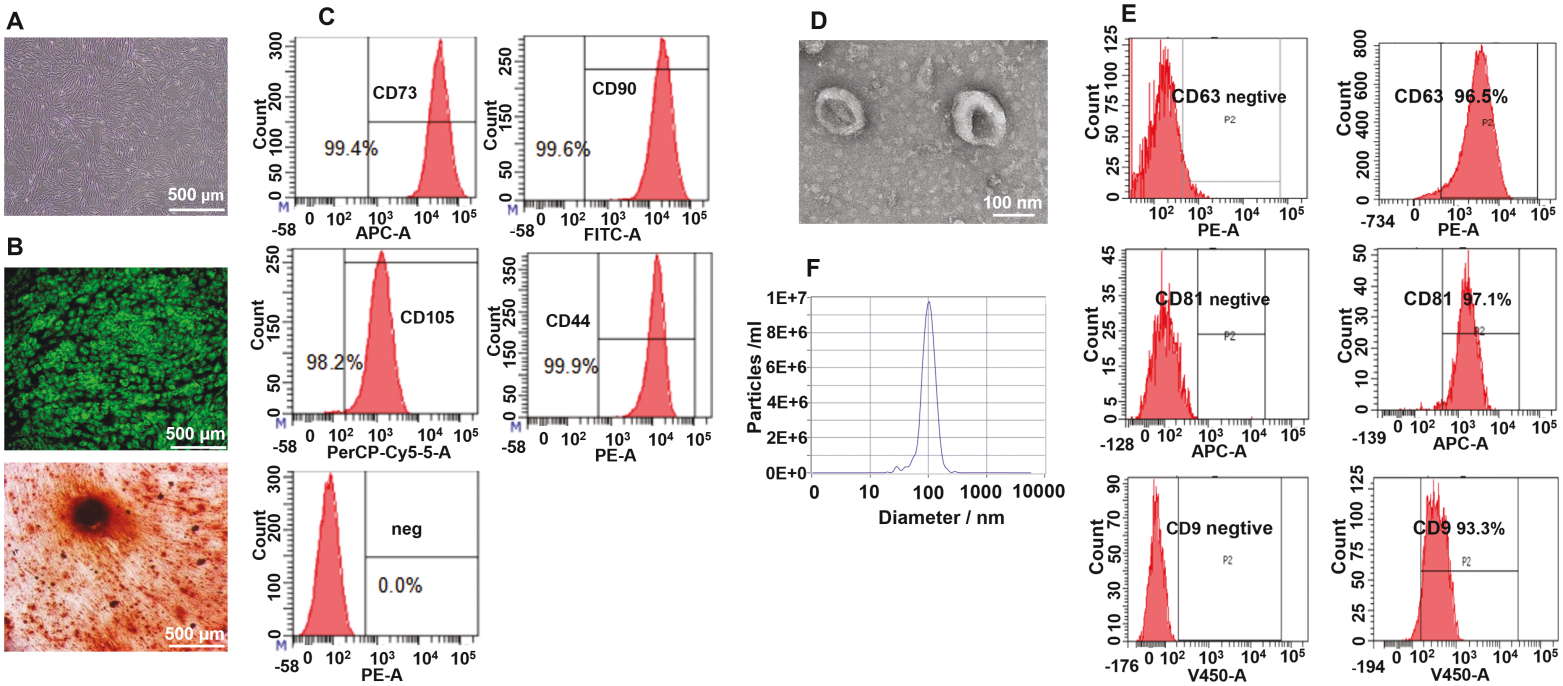
Characteristics of hUC-MSCs and hUC-MSCs-exosomes. (A) Microscope morphological observation of hUC-MSCs. (B) Adipogenic and osteogenic differentiation of hUC-MSCs. Cells rich in intracellular lipid droplets visualized with a fluorescent LipidTOX Green Neutral Lipid Stain indicate successful adipogenic induction. Chondrogenic and calcium phosphate mineral accumulation detected with 1% alizarin red after osteogenic induction confirmed the multipotent nature of hUC-MSCs. (C) Human UC-MSCs representative markers CD73, CD90, CD105, and CD44 were detected by flow cytometry analysis. The last panel is detected by a cocktail of negative control antibodies: CD34, CD45, CD11b, CD19, and HLA-DR negative antibodies mix. (D) TEM photograph of hUC-MSC-exosomes with negative staining using 2% uranyl acetate. (E) Exosome representative markers CD63, CD81, and CD9 were detected by flow cytometry analysis. Left panel of Figure E is negative isotype control. (F) Size distribution measurement of hUC-MSC-exosomes by NTA analysis.

Human UC-MSCs were isolated from the umbilical cord, and expanded in DMEM supplemented with 5% EliteGro-Adv. They were used between the fourth to sixth passages in subsequent experiments. The isolated hUC-MSCs showed the typical MSC morphology with spindle-like shape under the optical microscope (Figure 1A). Moreover, flow cytometer (Figure 1B) revealed that the isolated cells highly expressed the positive markers of stem cells (CD73, CD90, CD105, and CD44) and presented low level of CD34, CD11b, CD19, CD45, and HLA-DR (human MSC negative cocktail), indicating a highly enrichment of hUC-MSCs. After 24-hour culture in MSC complete medium, it was replaced with MSC adipocyte differentiation medium. After 7 days induction, adipogenesis was visualized by HCS LipidTOX Green neutral lipid stain (Thermo Fisher Scientific). Under adipogenic conditions, hUC-MSCs derivative stored triglycerides in lipid droplets as shown by HCS LipidTOX Green neutral lipid staining (Figure 1C). For osteogenic differentiation induction, MSC complete medium was replaced with MSC osteogenic differentiation medium for 12 days. Under the effect of osteogenic differentiation induction, mesenchymal stem cells (MSC) are gradually differentiated into osteoblasts, including calcium nodules formation and calcium secretion. Both of them can be dyed red by alizarin red S. Therefore, osteogenic differentiation was detected by alizarin red S staining. The hUC-MSCs differentiated into osteoblasts, as shown by alizarin red S staining (Figure 1C). These results indicate that the hUC-MSCs have multilineage differentiation capability.

The extraction of hUC-MSC-exosomes was performed by size-exclusion chromatography. The morphology of isolated hUC-MSC-exosomes was examined by TEM. The appearance of hUC-MSC-exosomes isolated matched the typical saucer-like shape (Figure 1D). Exosomal surface markers CD81, CD9, and CD63 were analyzed by flow cytometry. It showed that hUC-MSC-exosomes were positive for the exosomal surface markers CD81, CD9, and CD63 (Figure 1E). The ratio of CD81, CD9, and CD63 positive exosomes were, respectively, 96.5%, 97.1%, and 93.3%. To check the size homogeneity of hUC-MSCs-exosomes populations, we performed Nano Tracking Analysis and confirmed a homogeneous population of exosomes whose size was 103.1 nm and concentration was 9.8 × 10^6^ particles/mL (Figure 1F). These results indicated that the obtained hUC-MSC-exosomes confirmed to the well-established criteria.

### Human UC-MSC-exosomes attenuated OA progression

**Figure 2.**
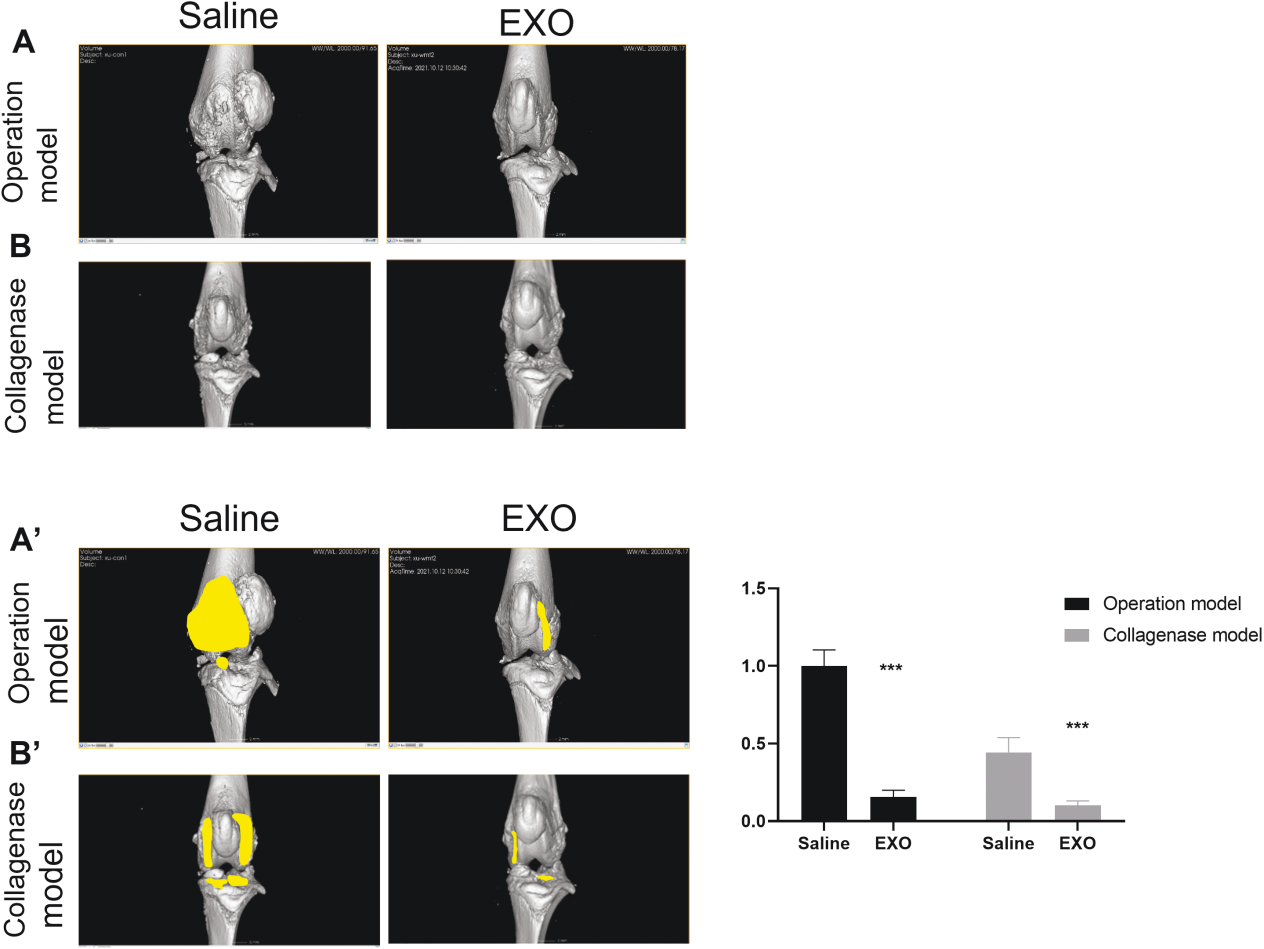
Micro-CT images of the knee joints of the OA animal models. The micro-CT images were reconstructed to 3D images. There are the saline group, underwent saline injection, and the exosome group, underwent hUC-MSCs-exosomes injection in (A) operation model and (B) collagenase model.

For the surgical model of OA, the left knee joints of rats were opened to expose the articular cavity, and the anterior cruciate ligaments, medial collateral ligaments, and medial menisci were cut off. For the collagenase-induced OA model, the collagenase was injected into the left hind knee joint cavity of the rats, and it was repeated on days 4 and 7 postinjection. These 2 OA models were randomly treated with saline (control group) and hUC-MSCs-EXO group with 5 × 10^9^ exosomes injection. Rat joints were scanned with the high-resolution micro-CT system and the images were reconstructed by the software Micro View to obtain micro-CT sections. Micro-CT images of the rat knees were used to characterize OA progression. Representative micro-CT 3D images of the OA operation and collagenase-treated rat models were shown (Figure 2A, 2B). In both of surgical model of OA and collagenase-induced OA model, the knee joint of the rats in the saline treatment group had a rough and irregular surface at the medial and lateral femur areas 4 weeks postsurgery. Compared with the saline treatment group, the knee joint in the exosome treatment group showed less loss and erosion of the cartilage in the medial tibial plateau and femur areas in 2 rat models (Figure 2A, 2B).

**Figure 3.**
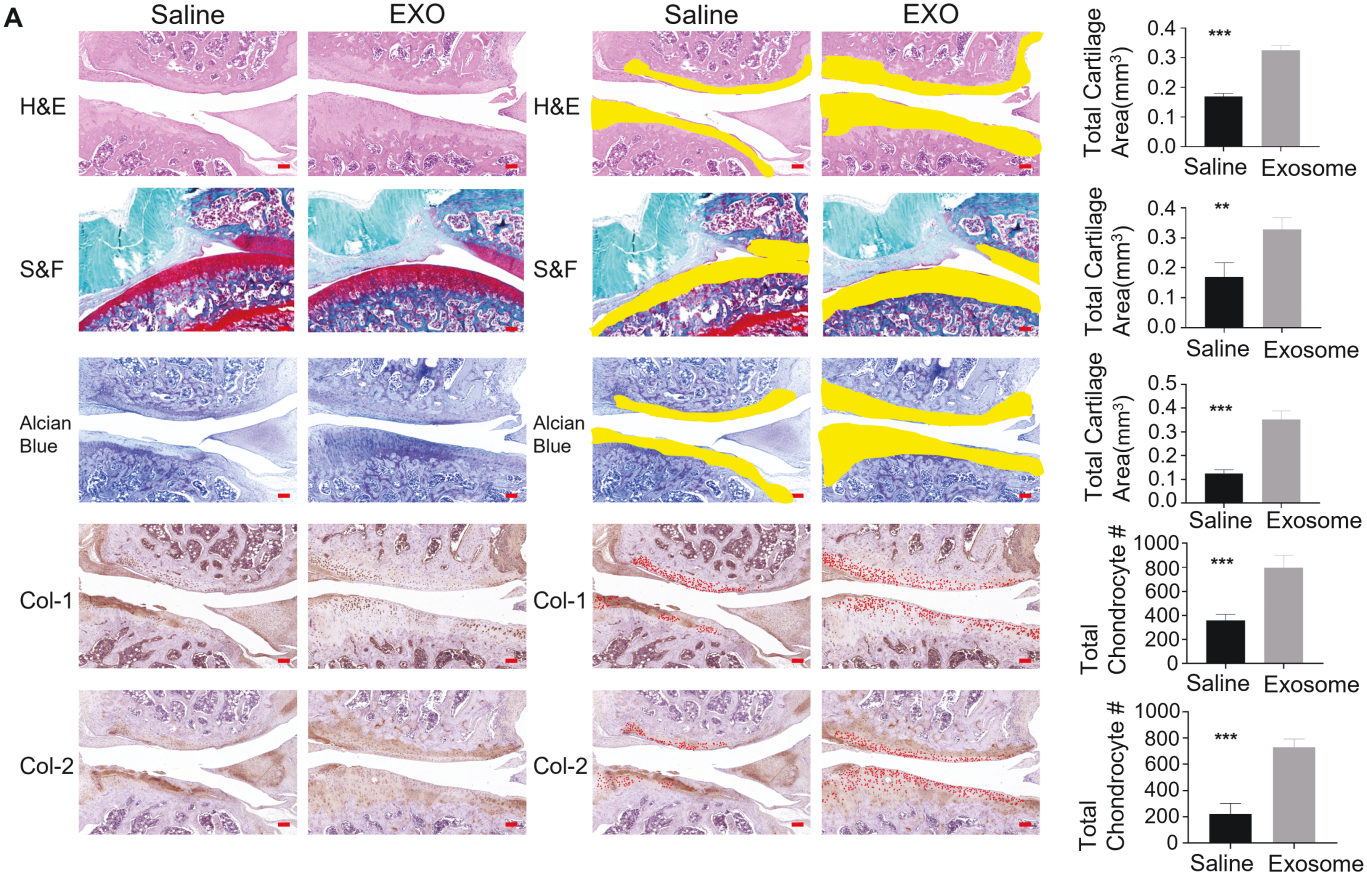

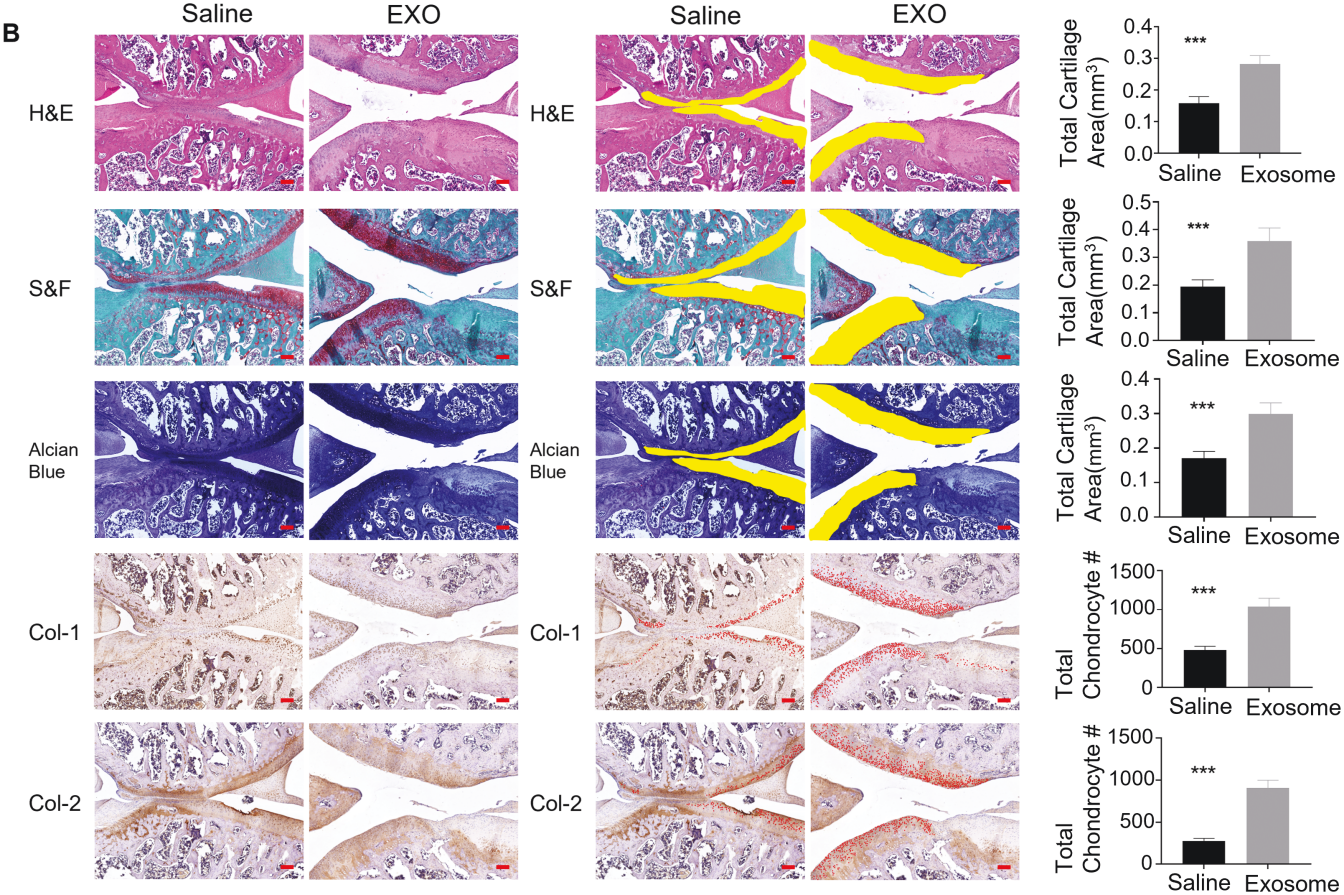
Histological evaluation of the knee joints of the OA animal models. Hematoxylin-eosin staining (H&E), Safranin O-fast green staining (S&F), Alcian blue staining, collagen-I staining, collagen-II staining was performed in (A) operation model and (B) collagenase model. (A) Each staining contained 2 groups in operation model: (1) operation model saline control group, underwent saline injection; (2) operation model exosome group, underwent hUC-MSCs-exosomes injection. (B) Each staining contained 2 groups in collagenase model: (1) collagenase model saline control group, underwent saline injection; (2) collagenase model exosome group, underwent hUC-MSCs-exosomes injection. In the left panel (saline and exosome columns in the left) of A and B, tibial articular surface stained with Hematoxylin-eosin staining, Safranin O-fast green staining, Alcian blue staining, collagen-I staining, and collagen-II staining in the control and exosome group. In the right panel (saline and exosome columns in the right) of A and B, histomorphometric analysis was used to trace the total cartilage area in the control and exosome group by the Hematoxylin-eosin staining, Safranin O-fast green staining, and Alcian blue staining, while collagen-I^+^ and collagen-II^+^ cells (red) were counted within the tibial articular tissue in the control and exosome group by the collagen-I staining and collagen-II staining. *n* = 3 rats per group. Bar = 100 μm.

The decalcification of knee joints was completed in 20% EDTA solution, and decalcified samples were dehydrated in graded alcohol solutions and embedded in paraffin. Slides were stained with hematoxylin/eosin, safranine O-/fast green, and Alcian blue. As shown in Figure 3A, 3B, H&E staining revealed that the joint cartilage of the saline treatment group showed noticeable degradation, including irregular superficial zone, degenerative matrix, and disarranged chondrocytes in both surgical models of OA and collagenase-induced OA model. In contrast, the articular cartilage of the exosome treatment group was smooth and intact in 2 rat models (Figure 3A, 3B). The basophilic cartilage tissue interacts with the alkaline dye safranine O to show red, while the eosinophilic bone tissue interacts with the acid dye solid green to show green. The green bone contrasts sharply with the red cartilage, thus distinguishing the cartilage tissue from the bone tissue. Alcian blue is an alkaline dye that stains acidic proteoglycans in cartilage. It specifically binds to the long chains of glycosaminoglycan sulfate present in the chondrocyte matrix and stained blue. Compared with the saline treatment group, the new cartilage of the exosome treatment group exhibited a more intense staining of safranin O and Alcian blue, and integrated with the adjacent cartilage in both rat models (Figure 3A, 3B). Immunohistochemical staining of collagen I and collagen II was performed to assess the extracellular matrix of newly formed bone and cartilage. The brown areas indicated positive expression of collagen I and collagen II in regenerated tissues. The visualization of osteogenic marker collagen I and chondrogenic marker collagen II is shown (Figure 3A, 3B). Collagen I and collagen II staining were located in newly formed bone and cartilage as detected histologically, respectively. The levels of collagen I and collagen II in the exosome treatment groups were higher than those in the saline treatment groups in both rat models (Figure 3A, 3B).

### Human UC-MSC-exosomes inhibited the production of synovial fluid cytokines and matrix-degrading enzymes

**Figure 4.**
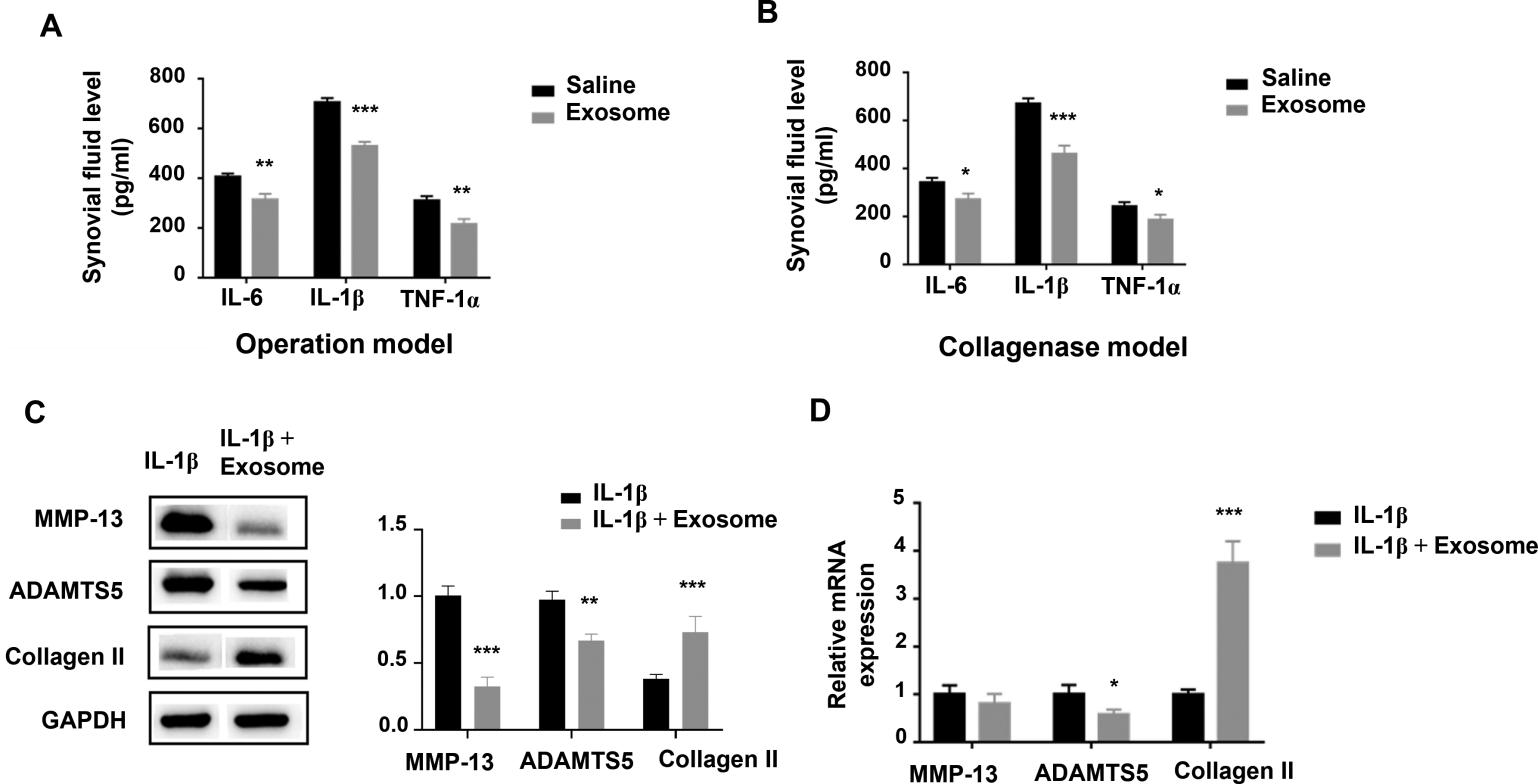
The change of synovial fluid cytokine (IL-1β, IL-6, and TNF-α) levels in rat knee joints and gene expression levels in IL-1β-stimulated chondrocytes with hUC-MSCs-exosome treatment. (A and B) The levels of inflammatory biomarkers IL-1β, IL-6, and TNF-α in synovial fluid were evaluated by ELISA in (A) operation model and (B) collagenase model. Bars represent mean ± SD. *P < .05, **P < .01, and ***P < .001 versus saline group; *n* = 3 rats per group. (C) The protein level of MMP-13, ADAMTS-5, and collagen II in IL-1β–stimulated chondrocytes were measured by Western blot. (D) The mRNA level of MMP-13, ADAMTS-5, and collagen II in IL-1β–stimulated chondrocytes were measured by quantitative real-time PCR. GAPDH was used as the internal control. Bars represent mean ± SD. *P < .05 and ***P < .001 versus control group; *n* = 3 per group.

The inflammation within the synovium plays an important role in the pathogenesis of OA. Proinflammatory cytokines, such as IL-6, IL-1β, and TNF-α, contribute to the destruction of cartilage and synovitis. The levels of synovial fluid cytokines including IL-6, IL-1β, and TNF-α were examined 4 weeks post the first treatment of rats with ELISA kits. The levels of inflammatory biomarkers in the synovial fluid, IL-6, IL-1β, and TNF-α in the exosome treatment group were lower than that in the saline treatment group in both 2 rat models, indicating an anti-inflammatory effect of hUC-MSCs-exosomes (Figure 4A, 4B).

Chondrocytes were isolated from the knee joint of rats and cultured in DMEM plus 10% FBS. Passage 1-3 chondrocytes were stimulated with recombinant human IL-1β for 48 hours and then treated with hUC-MSC-exosomes. Western blot was used to examine the protein expression of MMP-13, ADAMTS-5, and collagen in the primary chondrocytes. MMP-13 and ADAMTS-5 are matrix-degrading enzymes, and they promote cartilage degradation in OA. Exosome treatment significantly blocked IL-1β stimulated MMP-13 and ADAMTS-5 protein expression but promoted the expression of chondrogenic marker collagen II in chondrocytes (Figure 4C). Total RNA was also extracted from rat primary chondrocytes. The mRNA expression of MMP-13, ADAMTS-5, and collagen II in the primary chondrocytes was measured by qRT-PCR. The qRT-PCR results further demonstrated the changes in the expression of ADAMTS-5 and collagen II (Figure 4D). Especially, the expression of chondrogenic marker collagen II was dramatically increased in the exosome treatment group.

## Discussion

MSCs offer great potential for treatment of OA by promoting articular cartilage regeneration via paracrine secretion of exosomes. This study demonstrated that OA rats treated with hUC-MSC-exosomes had less morphological and histopathological cartilage damage and synovial membrane inflammation than rats treated with saline. Human UC-MSC-exosomes significantly downregulated the expression of MMP-13 and ADAMTS-5 and upregulated collagen II expression in IL-1β stimulated chondrocytes.

The levels of TNF-α, IL-1β, and IL-6 in the synovial fluid significantly decreased after hUC-MSC-exosome treatment in both OA rat models. TNF-α is a high-level cytokine that induces catabolism, allowing the generation of proteoclastic enzymes that destroy the cartilage. TNF-α is produced by activated monocyte and macrophagocyte, stimulates the osteoclasts gathering in the region of the topical bone resorption, leads inflammatory reaction, and contributes to removing topical minerals.^22^ IL-1β is known as a powerful cytokine that induces the dissolution of cartilage, produces inflammatory agents, such as prostaglandin E2 and nitric oxide, from the cartilage and synovial cells, and stimulates the expression of matrix metalloproteinases.^23^ It has been reported that IL-6 exhibits higher activity in the joint fluid than in the serum, suggesting an important role in OA pathology. IL-6 also facilitates the proliferation of synovial cells and increases the activity of osteoclasts, which form the pannus and produce proteoclastic enzymes that destroy the cartilage joint.^24,25^ Our results showed that hUC-MSC-exosomes had suppressed the inflammatory reaction of OA to prevent the loss and erosion of the articular cartilage.

IL-1β can upregulate cartilage matrix catabolic enzymes, including matrix metalloproteinases (MMPs) and ADAMTS-5, and inflammatory mediators PGE2 and NO in chondrocytes.^26,27^ MMPs are a class of proteinases responsible for the degradation of collagen II and proteoglycans in the articular cartilage, which play vital roles in extracellular matrix degradation in OA.^28,29^ MMP-13 is an important member of MMPs and its activity is elevated in human OA cartilage and experimental OA animal models.^30,31^ ADAMTS protein family is also implicated in cartilage degradation in OA, especially the ADAMTS-5.^8^ Therefore, MMP-13 and ADAMTS-5 are used as catabolic markers in OA. A previous study showed that MSC-exosome increased chondrogenic genes Col II and aggrecan, decreased MMP-13 and Runx2 in chondrocytes isolated from OA model mice and chondrocytes isolated from normal C57BL/6 mice treated IL-1β.^13^ They attenuated the proliferation inhibition and apoptosis induction of IL-1β-induced chondrocyte.^13^ In this study, UC-MSC-exosome treatment promoted cartilage regeneration in operation and collagenase-induced OA rat models. hUC-MSC-exosome treatment significantly also attenuated IL-1β-induced upregulation of MMP-13 and ADAMTS-5 in rat chondrocytes, which underlies its beneficial effects in protecting articular cartilage of the joints in OA rats.

## Conclusion

Human UC-MSC-exosomes were effective in treating operation and collagenase-induced rat models of OA. Specifically, hUC-MSC-exosomes significantly suppressed inflammation by inhibiting the production of proinflammatory cytokines, TNF-α, IL-1β, and IL-6. It was effective in preventing articular cartilage and synovial tissue degeneration. This suggests that hUC-MSC-exosomes have the therapeutic potential for the treatment of arthritis.

## Supplementary material

Supplementary material is available at *Stem Cells Translational Medicine* online.

szae031_suppl_Supplementary_Tables_1

## Data Availability

The data that support the findings of this study are available from the corresponding author upon reasonable request.
